# High Transmitter CD4+ T-Cell Count Shortly after the Time of Transmission in a Study of African Serodiscordant Couples

**DOI:** 10.1371/journal.pone.0134438

**Published:** 2015-08-20

**Authors:** Etienne Karita, Matt A Price, Shabir Lakhi, William Kilembe, Anatoli Kamali, Eugene Ruzagira, Eric Hunter, Paul Farmer, Susan Allen, Gwynn Stevens, Paramesh Chetty, Sabrina Welsh, Annie Yang, Jill Gilmour, Pat Fast, The IAVI Africa HIV Prevention Partnership

**Affiliations:** 1 Projet San Francisco, Kigali, Rwanda; 2 International AIDS Vaccine Initiative (IAVI), New York, New York, United States of America; 3 Zambia Emory HIV Research Project, Lusaka, Ndola and Kitwe, Zambia; 4 Medical Research Council/Uganda Virus Research Institute, Masaka and Entebbe, Uganda; 5 Emory Vaccine Center, Emory University, Atlanta, Georgia, United States of America; 6 School of Medicine, Emory University, Atlanta, Georgia, United States of America; 7 Human Immunology Laboratory, IAVI, London, United Kingdom; 8 UCSF Department of Epidemiology and Biostatistics, San Francisco, California, United States of America; University of Toronto, CANADA

## Abstract

**Background:**

2013 WHO guidelines recommend starting ART at CD4+ T-cell counts ≤500 cells/μL. We present the T-cell counts from adult Africans with HIV shortly following transmission to their sexual partners.

**Methods:**

HIV-discordant couples in Zambia, Uganda and Rwanda were followed prospectively and received couples counseling and condoms. HIV uninfected partners were tested for HIV at least quarterly and HIV-infected partners received HIV care and referral for ART per national guidelines. Upon diagnosis of incident HIV infection in the previously HIV-uninfected partner, a blood sample was collected from both partners to measure CD4+ T-cells and perform viral linkage. The estimated date of infection (EDI) of the incident case was calculated based on testing history. EDI was unknown for suspected transmitting partners.

**Results:**

From 2006–2011, 4,705 HIV-discordant couples were enrolled in this cohort, and 443 cases of incident HIV infection were documented. Virus linkage analysis was performed in 374 transmission pairs, and 273 (73%) transmissions were linked genetically. CD4 counts in the transmitting partner were measured a median of 56 days after EDI (mean:90.5, min:10, max:396). The median CD4 count was 339 cells/μl (mean:386.4, min:15, max:1,434), and the proportion of partners with a CD4+ T-cell count above 500/μl was 25% (95% CI:21, 31).

**Conclusions:**

In our cohort of discordant couples, 73% of HIV transmissions occurred within the relationship, and the transmitter CD4+ T cell count shortly after the transmission event was frequently higher than the WHO 2013 ART-initiation guidelines.

## Introduction

Approximately 69 percent of people living with HIV reside in sub-Saharan Africa (SSA), a region which continues to experience the highest HIV-1 incidence worldwide[1]. While 70 percent of all new HIV infections in 2010 were identified in SSA (accounting for 1.9 million new infections), increased access to antiretroviral therapy (ART) has played a role in decreasing the number of new infections and AIDS-related deaths in the region. HIV-1 replication is effectively suppressed in both blood and genital secretions when ART is taken, which decreases the likelihood of transmitting the virus to an uninfected partner [2–6]. In recent years, the correlation between ART use and the reduction of HIV-1 transmission has been strengthened by the results of a large clinical trial, modeling studies, and observational studies. A prospective cohort study of 3381 African heterosexual discordant couples provided evidence that ART significantly reduced the frequency of HIV-1 transmission events between partners[7]. HPTN 052 tested this hypothesis in a clinical trial, and found a dramatic reduction of HIV transmission among HIV-discordant couples where infected partners received ART [8]. Results from these and other large-scale clinical research provide strong evidence that the use of ART can significantly reduce the risk of onward HIV transmission [9–11].

The timing of ART initiation has significant implications for discordant heterosexual couples, who continue to be at very high risk of HIV infection in SSA [12, 13]. The latest update (June 2013) of the WHO guidelines on ART initiation recommends starting ART at a CD4+ T cell count (CD4 count) threshold of 500 cells/μL, an increase from the previous 2010 guidelines which advised initiating treatment at a threshold of 350 cells/μL [14]. The new guidelines also recommend initiating ART for persons in an HIV-discordant relationship regardless of CD4 count, and are predicted to expand the eligibility for ART initiation to an estimated 25.9 million people. This expansion, if achieved, could avert potentially 3 million AIDS-related deaths and prevent 3.5 million new HIV infections between 2013 and 2025. While the projected health gains appear to be significant, others in the field favor a universal coverage approach, or the “Test and Treat” strategy, which advocates the application of ART for all HIV-1 infected individuals regardless of CD4 counts [9]. The provision of ART at the level recommended by the WHO guidelines or via “Test and Treat” has important financial and logistical implications, especially in resource-poor settings.

The updated WHO guidelines propose covering an increasing proportion of persons with HIV which in turn will impact the onward transmission of HIV. However, without an understanding of the transmitter CD4 count at the time of transmission, it is challenging to estimate the proportion of transmission events that might be prevented by these new guidelines. Since 2006, IAVI has supported a cohort of consecutively identified volunteers with incident HIV infection from large HIV incidence, vaccine trial preparedness studies. This study presents the CD4 counts of genetically linked HIV-1 transmitters shortly after the time of transmission in an African cohort of HIV-1 discordant couples

## Methods

### Study population and clinical procedures

A prospective, multi-center cohort study enrolled and followed HIV discordant couples at 5 clinical research centers (CRC) in Rwanda (Projet San Francisco, Kigali), Uganda (Medical Research Council/Uganda Virus Research Institute, Masaka and Entebbe), and Zambia (Zambia-Emory HIV Research Project, Lusaka, Ndola, and Kitwe) to estimate HIV incidence and to describe predictors of seroconversion in the HIV-uninfected partner [15]. Volunteers were identified as HIV discordant in study clinic supported couples voluntary counseling and testing (CVCT), or were referred from government sponsored CVCT clinics; the majority of positive partners were newly diagnosed, no additional details on the duration of their infection was available.

At enrollment, both partners in the couple underwent a complete physical examination and a detailed medical history including onset of HIV-related illness and report of antiretroviral therapy (ART) if any, and a urine pregnancy test (female partner). Follow up was monthly or quarterly for HIV testing, risk reduction counseling, condoms, interim medical history and symptoms-directed physical examination as needed. Acute medical care at the study clinic or a referral slip for care and treatment at the nearby government clinic were provided as needed. Non-HIV STIs were managed syndromically or treated per national guidelines. If HIV care, including ART, was available near the CRC, study participants (both incident cases and suspected transmitting partners) were referred as needed per national ART initiation guidelines. Otherwise, HIV care, including ART, was provided by the CRC. These cohorts represent in part larger efforts to characterize cohorts that might be suitable for future HIV prevention trials [15]. As such, with the exception of initial work in Masaka, ART use in the HIV+ partner was a criterion for exclusion from the prospective incidence cohorts.

### Laboratory procedures

HIV testing included two rapid tests (Determine [Abbott Laboratories] and Unigold [Trinity Biotech], typically in parallel, and a p24 ELISA. Discrepant rapid test results were confirmed by a third rapid test or antibody ELISA. Upon detection of incident HIV infection, suspected transmitting partners were invited for a single blood draw for CD4 counts, viral load and sequencing. CD4 counts and viral load data were not available from the HIV positive partners prior to this visit. Laboratory testing including CD4 counts and routine laboratory tests were conducted at the CRCs; PCR [Roche Amplicor Monitor v1.5 (February 2006-January 2011) or Abbott Real Time HIV-1 v1.0 m2000sp/m2000rt, thereafter] for viral load (lower limit of detection: 50 copies/mL) was done at a central laboratory (CLS, Johannesburg, South Africa). All volunteers with incident HIV infection were confirmed by PCR; their last HIV antibody-negative sample was also tested by PCR to check for acute (i.e., Fiebig I) HIV infection at that visit. A positive syphilis test [rapid plasma reagin, Biotec Laboratories, Inc., UK] was confirmed by *T*. *pallidum* haemagglutination assay. Blood was collected for HIV viral load, sequencing and subtype determination. The REGA HIV-1 subtyping tool (http://hivdb.stanford.edu/) was used to analyze sequence from the *pol* gene from the first available specimen [16]. Additional sequencing or phylogenetic analysis was done if REGA results were indeterminate [17]. Molecular epidemiologic linkages were determined between volunteers with incident HIV infection and their suspected transmitting partner by sequence comparison of pol, gag, gp120, gp41, and/or long terminal repeat regions [18].

### Data and statistical analyses

Data analyses were conducted using STATA version 13 (Stata Corporation, College Station, Texas, USA). Data are presented as frequencies with percentages and 95% confidence intervals (CI). The estimated date of HIV infection (EDI) of the volunteer with incident infection was defined as either 1) the midpoint between the last negative and first positive HIV-antibody test, 2) 14 days before the first positive p24 antigen test in the absence of a positive antibody test, 3) 10 days before the first detectable viral load (VL) test in the absence of detectable p24 antigen or HIV antibodies, or 4) the date of a high-risk exposure event (i.e., a single, inter-visit report of sex without a condom, or condom breakage). All HIV-1 infection was confirmed by VL testing. The transmitting partner EDI was unknown; in this report EDI always refers to the estimated date of the transmission event between the transmitting partner and the volunteer with incident infection.

As CD4 counts tend to drop with time, we present the CD4 T cell data of linked partners in the entire cohort, and in that subset with data available within the 90 days of the EDI. The chi squared test or the Wilcoxon rank sum test are used to compare the proportion of epidemiologically linked transmission events and the subset of volunteers within 90 days of EDI by demographic characteristics. We used data on CD4+ T cell decline in African adults (at a low and high rate of 20 and 35 cells/year[19–21]) to estimate the transmitters’ CD4+ T cell counts at the time of transmission for both the subset with data within 90 days of EDI, and all transmitters.

### Ethics statement

All participants provided informed consent. Consent was documented; either by the volunteer’s signature or by his or her “mark” and the signature of an impartial witness if the volunteer was illiterate. This study was approved by: the Rwanda National Ethics Committee, the Uganda Virus Research Institute Science and Ethics Committee, the Uganda National Council of Science and Technology, the University of Zambia Research Ethics Committee, and the Emory University Institutional Review Board.

## Results

### Study Population

Between February 2006 and November 2011, 4,705 HIV discordant couples were enrolled and followed monthly or quarterly at five clinical research centers in Eastern and Southern Africa. During this period, 443 cases of incident HIV infection were documented, 241 (54%) in men and 202 (46%) in women. The HIV incidence rate ranged from 2.1 in Entebbe, to 10.8 per 100 person-years in Ndola (Table 1). Viral linkage analysis was performed in 374 (84%) couples, and 273 (73%) HIV transmissions were linked; this did not vary significantly by clinical research center (p = 0.09, Table 2). There were no statistically significant differences in sex, age, or level of education between linked and unlinked transmitting partners. Of those with linkage evaluated, 364 (97%) were married, 19 of whom were in a polygamous relationship. Marital status was significantly associated with viral linkage, with a greater proportion of monogamously married couples found to be linked (p = 0.01). We observed no statistically significant associations of demographic characteristics in linked transmitters with CD4 T cell data prior to 90 days post EDI compared to those with data >90 days post EDI (data not shown).

**Table 1:** HIV incidence and sex at each study site.

Study site	Enrolled couples	Female (%)	HIV incidence rate*
Kigali, Rwanda	1230	590 (48.0)	3.1 (2.5–3.6)
Masaka, Uganda	976	352 (36.1)	3.7 (2.8–4.6)
Entebbe, Uganda	598	322 (53.8)	2.1 (1.1–3.1)
Lusaka, Zambia	1150	522 (45.4)	M to F: 8.9 (7.8–10.0)
			F to M: 6.7 (5.9–7.7)
Ndola, Zambia	751	399 (53.1)	M to F: 10.8 (7.8–14.5)
			F to M: 6.1 (4.2–8.5)
Total	4705	2185 (46.4)	Range: 2.1–10.8

*Cases per 100 person-years (95% CI). Stratified by sex only when the difference was statistically significant (p<0.05)

For more details, see [15]

**Table 2:** Demographic characteristics of the suspected transmitting partners of incident cases reported to be in HIV discordant couple relationships.

		Linkage status				
Transmitting partner characteristic	Total	Linked	%	Unlinked	%	p value ▪
Total	374	273	73.0	101	27.0	
Sex						
Male	164	124	75.6	40	24.4	0.31
Female	210	149	71.0	61	29.0	
Clinical Research Center						
Kigali	95	75	79.0	20	21.0	0.09
Masaka	63	42	66.7	21	33.3	
Lusaka	133	95	71.4	38	28.6	
Entebbe	12	12	100.0	0	0.0	
Kitwe & Ndola	71	49	69.0	22	31.0	
On ART (self report) at EDI**						
Yes	5°	1	20.0	4	80.0	0.007
No	369	272	73.7	97	26.3	
Age at enrollment						
Median	32	32		32		0.88
Mean	33.0	33.0		33.0		
IQR	27–37	27–37		27–37		
Marital status						
Single	4	1	25.0	3	75.0	0.01
Previously married▪▪	6	4	66.7	2	33.3	
Married, monogamous	341	256	75.1	85	24.9	
Married, polygamous	23	12	52.2	11	47.8	
Educational achievement at enrollment						
None	42	31	73.8	11	26.2	0.80
Some primary	231	168	72.7	63	27.3	
Some secondary	88	63	71.6	25	28.4	
More than secondary	13	11	84.6	2	15.4	

* Data or sample not available from one or both volunteers

** Transmitting partner reports use of antiretroviral therapy, EDI: Estimated date of infection in the incident case

°An additional 6 volunteers reported being on ART at or around the time of the transmission event, however their viral load was too low to sequence for linkage analysis

^▪^ Comparing linked vs. unlinked across volunteer characteristic

^▪▪^ Divorced, separated or widowed

### CD4+ T cell count in the transmitting partner

CD4 counts in the 273 linked transmitting partners were measured soon after the transmission event was documented (1/273 (<1%) linked partners did not have CD4+ T cell data). The median CD4 count was 339 cells/μl (mean: 386.4, min: 15 max: 1,434) and 134 of the 272 transmitting partners (49%; 95% CI: 43, 55) had CD4 counts above 350, whereas 69 (25%; 95% CI: 21, 31) had CD4 counts above 500 (Table 3). Considering that the time interval from the EDI to CD4 cell measurement ranged from 10 to 396 days (median: 53, mean: 86.6), and assuming an annual decline in African adults at 20–35 cells/year [19–21], we estimated the median CD4 count at the time of transmission at 350–356 cells/μl, and the percent of transmitting partners with CD4 counts above 500 at 26% (71 vs. 72 transmitters above 500 cells, respectively).

**Table 3:** CD4 counts (cells/μL) in 272 linked transmitters with data shortly after transmission showing total cohort and stratified by time since estimated date of transmission event.

	N	%	Median	Mean	Range
Total	272	100	339	386.4	15–1,434
>350 cells	134	49			
>500 cells	69	25			
≤90 days*	193	100	382	420.3	34–1,218
>350 cells	110	57			
>500 cells	60	31			
>90 days*	79	100	282	303.7	15–1,434
>350 cells	24	30			
>500 cells	9	11			

*Data collected ≤ or > 90 days post the estimated date of the transmission event

**Fig 1:**
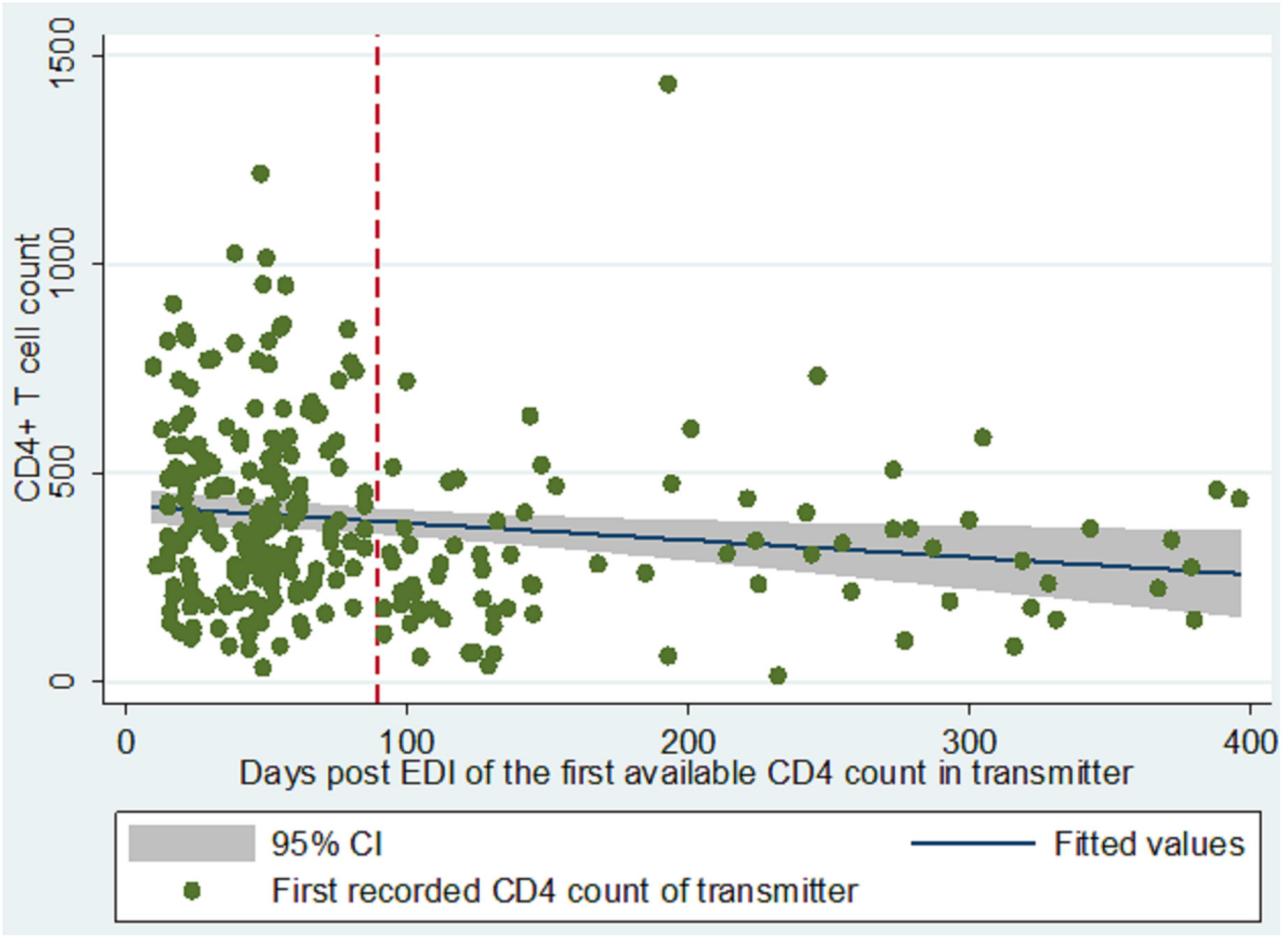
First available CD4 count in 272 linked transmitting partners following HIV transmission. Vertical dashed line shows the analysis cut point of 90 days post EDI.

CD4 counts tended to be lower as the number of days post EDI increased (Fig 1): the median CD4 counts among 193 linked partners enrolled during the first 90 days post EDI was 382 (mean: 420.3, range: 34–1,218) compared to 282 (303.7, 15–1,434) among those enrolled after 90 days (p = 0.0001). If we consider only the 193 partners who were enrolled during the first 90 days after the EDI, the number of transmitting partners with CD4 counts above 350 cells/μl was 110 (57%, 95% CI: 50, 64), and that of partners with CD4 counts above 500 cells/μl was 60 (31%, 95% CI: 25, 38). Using data from this group, our estimate of CD4 counts increases to a median of 385–386 cells at the time of transmission, with the percent above 500 cells at 32% regardless of the estimate for decline used (61 vs 62 transmitters above 500 cells, respectively).

### Viral load and reported ART use at EDI and enrollment

Viral load data were available from 253 (93%) of the linked transmitters, 10 of whom were undetectable due to ART initiation. These 10 transmitters had initiated ART after the transmission event, but prior to enrollment in this study; viral linkages were done on older, archived samples. Viral load among the 243 linked transmitters with detectable values was typically measured at the same visit as the CD4 counts; the median viral load was 69,500 copies/mL (IQR: 17,900–196,000, range: 208–8,520,000). Linked transmitters with CD4 counts of 500 and below had significantly higher median viral load values than those with higher CD4 counts (102,500 copies/mL [Interquartile range: 28,750–216,000] vs. 17,800 copies/mL [IQR: 6,767–79,900] respectively, p<0.001). This relationship was similar among transmitters presenting with CD4 counts at or below 350 cells/μl also having higher median viral loads compared to those with higher counts (115,000 copies/mL [IQR: 38,700–275,000] vs. 41,865 [IQR: 8,930–125,000], respectively, p<0.001).

ART was typically an exclusion criterion for discordant couples in the incidence cohorts, except from 2006–2009 in Masaka, Uganda. Once an HIV+ partner went on ART (including after 2009 in Masaka), the couple was taken off study at a subsequent visit, however due to reporting delays this was not always at the visit immediately following ART initiation. Of the 399 transmitting partners enrolled, 11 (3%) reported ART use starting an average of 170 days (median; mean: 216, min: 25, max: 855) prior to the recipient’s EDI. Sequencing was not possible in the majority (6) of these volunteers due to viral suppression; however in the remaining 5, only 1 (20%) transmission was linked to their partner (p = 0.007, Table 2). This partner started ART 225 days before the EDI, and appeared to be failing treatment with a VL of 489,000 c/mL 24 days after the EDI.

## Discussion

In this study of HIV transmission among African HIV discordant couples, we present data on CD4 counts in the chronically infected partners shortly after they transmit the virus to their sexual partner. The findings from the study suggest that, close to the time of transmission, more than 50% of the transmitting partners have a CD4 count of >350 cells/μl, and approximately one third have a CD4 count of >500 cells/μl, despite the fact that these transmitters with higher CD4 counts also tended to have lower viral loads. Similar results were found in the Partners in Prevention HSV/HIV Transmission Study, in which 51% of transmitting partners had a CD4 count of >350 cells/μl, and 28% had a CD4 count of >500 cells/μl (7).

The 2013 WHO guidelines on the use of ART for HIV treatment and prevention recommend initiating ART at a CD4 threshold of 500 or fewer cells/μL, with priority given to those with CD4 counts ≤350. The guidelines also recommend putting all HIV-infected partners within HIV discordant couples on ART, regardless of their CD4 counts, to reduce risk of transmission to the uninfected partner. The implementation of these guidelines will be challenging, particularly in Sub-Saharan Africa, the region where the highest prevalence of HIV discordant partnerships is found [12]. Many adults have not yet been tested for HIV and do not know their HIV status or the HIV status of their partner. In a previous report from this cohort, we reported that CD4+ T cell counts fall below 500 in a majority of volunteers within 9 months of EDI [22]; given the delay between infection and diagnosis that is so common with HIV infection, these new guidelines may be functionally similar to a “test and treat” program as nearly everyone who tests positive for HIV may be eligible for ART at the time of their diagnosis. Financial constraints and limited health care infrastructure also limit access of people living with HIV to care and treatment programs; in many cases, priority is given to those with advanced HIV disease and/ or very low CD4 counts [23]. It is also important to note that as of today, relatively few programs in Africa have adopted CVCT as standard of care for the prevention of HIV transmission [24], and these services, in many cases, have only been offered in the context of research projects [25, 26]. More financial resources will therefore be needed to strengthen the health infrastructure, to promote CVCT services, and to procure more HIV test kits and ART drugs.

The acceptability of treatment initiation and high adherence to ART will be critical to prevent onward transmission, particularly for those who will be requested to initiate treatment while they perceive themselves as still healthy and have high CD4 counts. HIV infected persons who feel healthy may be less likely to get tested. Even if tested, they may be less likely to be eligible for ART and more likely to drop out of pre-ART care [27]. Because of this, ART refusals or treatment failure due to feeling healthy remain challenging to measure and data remain sparse. One study conducted in Ethiopia reported on 551 persons (18%) who dropped out of a cohort of 3,012 persons starting on ART. The authors found 48% of drop outs were due to deaths, 12% were untraceable, and among the remaining 135 who stopped ART, nearly a third (41) did so because they perceived themselves as improved, a further 75 (56%) reported stopping as they preferred traditional medicine to the ART [28].

Another important finding that our study reinforces is the prevalence of unlinked transmissions. While the 2013 WHO Guidelines recommend initiating ART in the positive partner of a discordant couple, our study finds that approximately one quarter of transmission events occur outside of the established relationship. Previous studies have documented rates ranging between 15% and 30% [18, 29, 30]. In the HPTN 052 clinical trial of ART as prevention, 28% of transmission events were not linked to their reported sexual partner [8]; while the efficacy of this intervention was clearly very high, the effectiveness in the real world will likely be considerably lower due to these transmission events from outside the couple.

A limitation of our study includes the delay between the transmission event and measurement of CD4 counts in the transmitting partners. We collected the information on CD4 count and viral load in the suspected transmitting partners only after the transmission event was documented, typically one to three months after the estimated date of infection (median of 58 days). It is possible that some transmitting partners observed higher than expected T cell count drops during this interval, and also that our viral load data may not be ideal representations of the data at the time of transmission. However, CD4 T cell decline is expected to be modest in this interval [19–21], and there was no significant difference between the observed proportion of partners with CD4 counts above 500 and that estimated by modeling. Additionally, our study focuses on discordant couples, which represent one of the largest at-risk groups in Africa [12, 13]. While we observed that approximately one third of transmission events were from a donor with a CD4 count above 500, it’s not clear that this can be generalized to other epidemiologically distinct key populations such as sex workers or men who report sex with men. However, observing transmission pairs in many key populations can be considerably more challenging.

In summary, our study shows that a significant proportion of HIV transmission occurs above the current CD4-based eligibility guidelines while the index partner may still be healthy and less inclined to adhere to treatment. A significant proportion of transmissions also occurs through sex with persons other than one’s regular partner. These findings reinforce the importance of strengthening prevention of onward transmission in HIV infected individuals through early diagnosis, treatment and linkage to care. Regular testing of couples together followed by prompt treatment of the HIV infected partner is an efficient and workable solution to minimizing the number of persons who are unknowingly in jeopardy because their spouse is HIV infected.

## Supporting Information

S1 File Study data supplementary material file #1. (CSV)

S2 File Study data supplementary material file #2. (TXT)
